# Target Validation Studies of PS48, a PDK-1 Allosteric Agonist, for the Treatment of Alzheimer’s Disease Phenotype in APP/PS1 Transgenic Mice

**DOI:** 10.3390/ijms26083473

**Published:** 2025-04-08

**Authors:** Henry W. Querfurth, Cynthia Lemere, Jason Ciola, Daniel Havas, Weiming Xia, Han Kyu Lee

**Affiliations:** 1 Tufts Medical Center, Department of Neurology and Tufts University School of Medicine, Department of Neuroscience, 800 Washington St., and 136 Harrison Ave., Boston, MA 02111, USA; 2 Brigham and Women’s Hospital, ARCND, 60 Fenwood Rd., Hale Bldg. for Transformative Medicine, Boston, MA 02115, USA; clemere@bwh.harvard.edu (C.L.); jciola@bwh.harvard.edu (J.C.); 3 Psychogenics Inc., 215 College Rd., Paramus, NJ 07652, USA; daniel.havas@psychogenics.com; 4 Chobanian and Avedisian School of Medicine, Department of Pharmacology, Physiology and Biophysics, Boston University, 72 E. Concord St., Boston, MA 02118, USA; wxia@bu.edu; 5 Tufts Medical Center, Department of Neurology, 800 Washington St., Boston, MA 02111, USA; hlee@tuftsmedicine.org

**Keywords:** Alzheimer’s disease, insulin resistance, transgenic mice, PDK-1 agonist PS48, glycogen synthetase kinase, Tau phosphorylation

## Abstract

The Alzheimer’s disease (AD)-affected brain is known to be deficient in the utilization of glucose, its main energy substrate, and systemic diabetes is a significant risk factor for AD. In the course of biochemical and molecular investigations into this puzzling relationship, it has been shown that resistance to insulin action is a prominent feature of early stages of AD in the brain, thereby contributing to an energy failure state and a decline in synaptic function. In one AD-like cellular model, we found that β-amyloid (Aβ) accumulation inhibited insulin signaling and cell viability through an alteration of the PI3K/PDK-1/Akt signal pathway, an effect overcome by mTORC2 stimulation. A PDK-1 allosteric agonist, PS48, as well as newly synthesized analogs, were also found to reverse the metabolic defects caused by intracellular Aβ42 accumulation. In vivo, we previously showed that oral dosing of PS48 significantly improves learning and memory in APP/PS1 transgenic mice. Herein, we present evidence using unbiased immunohistological quantification and Western blot analyses demonstrating that ingested PS48 crosses into brain tissue where it targeted Akt and GSK3-β activities. Beneficial effects on neuronal number and Tau phosphorylation were found. Not unexpectedly, Aβ levels remained unchanged. These results support a path toward a future therapeutic trial of this untested strategy and agent in humans.

## 1. Introduction

The relationship of peripheral insulin resistance (IR) and diabetes mellitis type 2 (DM2) to sporadic Alzheimer’s disease (sAD) has been studied for over 25 years using epidemiological, genetic, radiologic, cell biology, and molecular techniques. Systemic diabetes, among other acquired factors, carries a relatively high risk of association with either AD or all-cause dementia. Relative risk (RR) and odds ratios (ORs) range from 1.5 to 2.8 [1,2,3,4]. This alone would account for a population attributable risk (PAR) for AD in the U.S.A. of 4.5% [5]. Having the full metabolic syndrome with comorbidities that include hypertension, diabetes, obesity, and inactivity increases the PAR to ~41% [5]. The risk attributable to DM is comparable to that of harboring a single apolipoprotein E4 allele (ApoE4), the strongest genetic-based risk factor, for which RR and OR reports range from 2.0 to 3.2 and PAR reports are as high as 50% [6,7,8]. Moreover, deficient insulin metabolism, whether from resistance and/or hypoinsulinemic responses can interact with ApoE4 in the pathogenesis of AD [9]. Thus, diabetes with an ApoE4 allele carries an RR of 5.5 for AD, relative to having neither [10]. Having an abnormal Homeostatic Model Assessment-Insulin Resistance index (HOMA-IR) in midlife is additive with ApoE4, yielding a hazard ratio of 1.55 for AD in late life relative to no ApoE4 [11]. Moreover, ApoE4 allele presence and abnormal HOMA-IR independently and additively reduce brain glucose uptake but do not interact [12]. Nor does ApoE4 associate with biochemical measures of brain insulin signaling in diabetics [13]. Notwithstanding the biologic pathways that may link these two, whether interacting or simply additive, the AD brain itself is in a diabetic state, both deficient in and resistant to central and peripherally produced insulin and insulin-like growth factor 1 (IGF-1).

The explored mechanisms behind defective insulin signaling in the AD brain have included each of the following ordered steps leading to Akt (protein kinase B, PKB) activation [14,15]: decline in insulin receptor (IR) number, decline in ligand affinity, tyrosine kinase deactivation, inhibitory phosphorylation of insulin receptor substrate (IRS-1, via p70S6K), reduced PI3K (p85) activation, and impairment of the coordinated PDK-1- and mTORC2-dependent Akt-1 activation sequence resulting in T308 and S473 phosphorylations of the latter, respectively [16,17]. The consequences to the neuron of a deficiency in this signal output include the following: derepressed autophagy, apoptosis, release from cell cycle arrest, deficient DNA repair, reduced ribosome biogenesis/protein translation, and defective glucose metabolism/GLUT3/4-mediated transport. Several of these negative outcomes result from the loss of both Akt-mediated, positive regulation of mTORC1(indirect) and Akt-driven inhibition of glycogen synthetase kinase (GSK-3α/β via phosphorylation on S9 (direct)).

GSK3α/β plays critical roles among the many signaling kinases and cofactors implicated in AD neurodegeneration, usually acting to negatively regulate its substrates through phosphorylations. These include the following: suppression of Wnt signaling by sequestering β catenin, downregulation of mitochondrial biogenesis and metabolism by inhibiting PGC-1α, and suppression of LTP-driven synaptic plasticity by phosphorylating CREB [18]. Pertinent to our work, active GSK antagonizes insulin signaling by inhibiting mTORC2 (Rictor, a stimulatory Akt-kinase), in addition to suppressing mTORC1 and promoting ULK-1-mediated autophagy. Furthermore, GSK3β is a major Tau kinase, phosphorylating many epitope sites including the following: AT180 (T231, S235) and AT8 (S202, T205) in the proline rich region as well as PHF1 (S396, S404) in the C’ region [19,20]. Hyperphosphorylation of Tau by disinhibited GSK has negative effects on axonal transport and synaptic plasticity [21].

Many strategies to improve insulin signaling and/or deactivate GSK have been tested in clinical MCI/AD trials. These have included the following: the incretin hormone, GLP-1 agonists, semaglutide, liraglutide and dulaglutide (NCT04777396, NCT01469351, NCT 01394952) [22,23], inhibitors of the glucose co-transporters, dapagliflozin and empagliflozin (NCT05081219) [24], as well as Pioglitazone [25], Tideglusib, a GSK inhibitor [26], and intranasal insulin [27]. Many of these phase II and III trials proved ineffective or were terminated after showing early promise in preclinical and phase 1 tests. Nonetheless, results from the REWIND trial of dulaglutide have shown promise in reducing cognitive decline in >50 year old patients with diabetes who were followed over 5 years (HR 0.86) [28]. A read-out from the two large ongoing phase III EVOKE trials of semaglutide in MCI and early AD are expected in 2015 (NCT04777396). To our knowledge, there are no clinical trials in progress that test a strategy to directly restore insulin sensitivity at the Akt-1 level of signal transduction.

The absent clinical effort to target Akt in AD may in part be related to conflicting assessments of basal Akt activation status, as is usually quantified by proxy phosphoAkt ratio determinations. In brain samples from AD subjects or diabetics with AD neuropathology, such ratios show either abnormally high [13,29,30,31,32] or reduced [33,34,35,36,37] levels. In one seminal work, all phospho-ratios of kinases (ERK, mTORC1, S6K, IKK, JNK1/2, and Akt) were found elevated in the AD brain [38]. Nonetheless, they all turned out to be insensitive to their normal stimulation by insulin, e.g., from IRS-1 deactivation. Therefore, we contemplated a strategy to restore Akt sensitivity, without overstimulation and its theoretical risk of oncogenesis.

We first noted attempts to model the interaction of AD pathology with systemic insulin resistance and obesity using APPswe transgenic mice (line 2576), to show an aggravation of β-amyloid (Aβ) pathology when reared on a high-fat diet [39]. The mice evidenced inhibited brain Akt-1 and derepressed GSK3β. Our model of disrupted insulin signaling was confirmed in vitro with cultured neurons induced to express cellular Aβ using viral vectors to show that normal insulin-mediated stimulation of Akt was blocked by ~40% by the Aβ expression [37]. The result was confirmed in both an Akt enzymatic in vivo/in vitro assay and a reconstituted reaction system.

Next, we tested a novel small-molecule allosteric agonist of PDK-1, termed PS48 [40]. PDK-1 is an S-T kinase of the AGC class [41], essential to the activation of Akt [15,42]. PS48 reversed the negative effects of intracellular β-amyloid accumulation on insulin signaling by restoring Akt T308 phosphorylation and activity, neuronal cell viability, and synaptic plasticity. The compound was active in the 10 nM range [43]. We then used a double transgenic APPswe/PSEN-1dE9 mouse model line (4462) of Alzheimer’s disease amyloidosis to test the effect of oral administration of PS48 (50 mg/Kg/d) on preventing the expected decline in learning and memory in the Morris Water Maze (MWM) spatial paradigm. We assessed the drug’s effectiveness to preserve cognition in transgenic mice raised on a standard diet and on a high-fat diet in order to further impair central insulin signaling [44]. Our hypothesis is that the cognitive improvement we witnessed in AD mice raised on either or both diets and treated with an indirect Akt agonist would be reflected in restored central Akt activation and GSk-3β inhibition. Here, we validate that the drug effectively penetrated the blood–brain barrier to affect its intended molecular targets in the relevant memory and learning prefrontal and hippocampal brain regions. Evidence is presented for the reversal of pAkt, pGSK, and pTau abnormalities in the dosed transgenic animals by measures of enzyme activity, protein fractionation, and immunofluorescence.

## 2. Results

Drug-target validation studies were carried out on groups of wild-type (WT) and transgenic (TG) mice bearing a set of familial Alzheimer’s disease-causing mutations, raised either on a standard diet (SD) or high-fat diet (HFD). The latter dietary intervention results in a metabolic syndrome (weight gain and dysregulation of fasting and OGTT glucose control) that correlates with a worsening of the transgenic AD-like cognitive phenotype, already shown to consist of spatial learning and memory decline [44]. Out of the original cohort of 36 animals that were cognitively tested, we performed Western blot analysis and immunohistology on 25, divided into five equal sized groups: (1). Wild-type littermates fed a standard diet with added vehicle (WT/SD, n = 5) serving as a baseline control to the other four groups (all transgenic); (2). TG/SD/V (n = 5); (3). TG/SD/PS48-treated (n = 5); (4). TG/HFD/V (n = 5); and (5). TG/HFD/PS48 (n = 5). The mouse brains were harvested at 62 weeks of age, after 18 weeks of oral drug treatment ending with the cognitive test phase.

### 2.1. Immunoprecipitations: Western Blot and Kinase Activity Analysis

**Figure 1:**
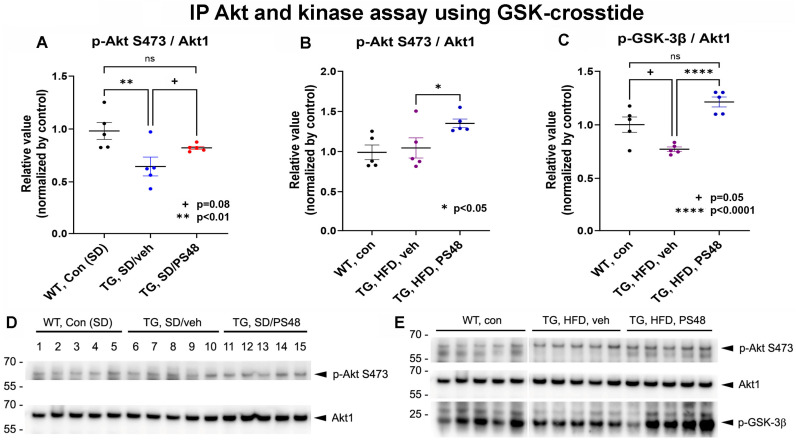
**Immunoprecipitation (IP) of Akt-1 and kinase activity.** (**A**) Standard diet (SD) condition. Tissue lysates were prepared from hemibrain temporal cortex and hippocampus of wild-type (WT or control) and transgenic (TG) mutant APP/PS1 adult mice (18 mo. of age) that excluded prefrontal, brainstem, and cerebellar regions. These were incubated with mouse mAb SC-5298 to IP total Akt. Compared to WT mice, TG mice raised on a SD and dosed vehicle (DMSO) showed significantly reduced (~35%) phosphorylated AKT as detected using anti pAKTS473 (corrected for total Akt and normalized to WT, *p* < 0.01). PS48 in the diet partially rescued pAKT to a mean level higher than the TG/veh, i.e. not exposed to PS48, basal level. Although the difference from the basal level did not reach significance (*p* = 0.08), the treatment level approached the WT control from which it was not significantly different (ns). (**B**) High-fat diet (HFD) condition. Method was as above and included kinase activity (phospho-consensus GSK3β). The TG animals on an HFD supplemented only with the vehicle showed no change in pAkt levels compared to WT (Control). However, PS48-fed animals showed elevated pAkt levels relative to WT and TG/veh (*p* < 0.01 and *p* < 0.05, respectively; n = 5). (**C**) Akt activity assessed by phosphorylation of a GSK consensus sequence (crosstide). In TG animals fed an HFD, Akt activity was depressed, albeit short of significance (*p* = 0.05), compared to WT on a SD. Note also the variance with the pAkt result in (**B**) that showed no change. TG on an HFD exposed to PS48 showed a significant rebound in activity (*p* < 0.0001) compared to the vehicle control and also to a higher level than the WT control (ns), noting the similarity to A. The pGSK signal was normalized to IP total Akt. (**D**) Representative Western blot of the IP reaction for total Akt and pAKTS473 (Standard diet, n = 5 of 8 animals each group). (**E**) Representative Western blot of the IP reaction for total Akt and pAKTS473 (High fat diet, n = 5 of 8 animals). Phosphorylated crosstide results shown below.

We first sought protein-based evidence that the study drug acted on its intended target in the brain. The expected site of PS48 action, an allosteric PDK-1 agonist, would be Akt-1 activation or at least restoration from inhibition as shown in a previous work [43]. We prepared brain homogenates of the cerebrum anterior to the mid colliculi but caudal to (excluding) the olfactory bulb, striatum, and sensorimotor and orbitofrontal areas. Thereby, the hippocampal formation, thalamus, amygdala, and visual area were isolated. Total Akt-1 was immunoprecipitated (IP) and then fractionated by Western blot to detect its activated phosphoS473 Akt-1 (pAkt) form. After densitometric measurement of the signals, statistical analysis of the derived ratio of pAkt/total Akt showed a significant group effect (1-way ANOVA; F(2,12) = 5.8, *p* = 0.01). This revealed a significant reduction (~35%) in Akt activation in the TG/SD/V animals compared to the WT/SD control (*p* < 0.01, n = 5 ea) Figure 1A. Treatment with PS48 partially corrected the loss. While not quite reaching significance compared to basal inhibition (*p* = 0.08), treatment yielded a level near equal to baseline WT (ns, not statistically significant). Although TG animals on HFD showed no reduction in pAkt/totAkt, PS48 nevertheless significantly increased activating phosphorylation to a level exceeding the control (*p* < 0.05, n = 5) (ANOVA summary; F(2,12) = 4.2, *p* = 0.04) Figure 1B.

In straight Western PAGE of whole-tissue lysates, consistent trends in TG animals fed SD were noted, evidencing a slight depression of the pAkt/Akt ratio compared to WT which then rebounded with the addition of PS48 (ANOVA; F = 0.95(2,12), *p* = 0.41, ns) (Supplement Figure S1A). A trend to deactivate (inhibitory phosphorylate) endogenous glycogen synthetase kinase-3β/α (GSK), a 47–51 kDa S/T kinase downstream substrate of Akt, by PS48 was found (ANOVA; F(2,12) = 1.44, *p* = 0.27, ns) (Supplement Figure S1B). The small increase in the pS9GSK-3β/tot GSK protein ratio alongside the pS473Akt/tot Akt change in animals fed PS48 on a SD suggests their functional link following PS48-mediated recovery of Akt activity (as shown in the IP Akt experiments). In a separate experiment on TG animals fed an HFD, whole-brain extracts from animals exposed to PS48 showed a significant increase in pAkt/tot Akt relative to the vehicle control (*p* < 0.05). However, the same animals revealed no change in endogenous pGSK with treatment (Supplement Figure S1C).

Our past experience using whole-cell culture extracts in direct Western blots for pAKT detection (as a proxy of activation) demonstrated variance in results that were either statistically significant or ‘trending only’. We then found Akt enzymatic activity level determinations to be a more reliable assay. When the IP Akt was added to an in vitro reaction with an added GSK3-β consensus peptide-fusion (crosstide, MW 20 kDa) as a substrate, we saw that Akt activity in TG mice was suppressed by ~25%, just short of significance (*p* = 0.05). Under these HFD/vehicle conditions, the inhibition was convincingly reversed by treatment with PS48 (*p* < 0.0001, n = 5), to a level statistically on par with WT (ns) (summary ANOVA; F = 18.8(2,12), *p* = 0.0002) Figure 1C.

### 2.2. Immunohistochemistry: Regional Size and Neuron Number

**Figure 2:**
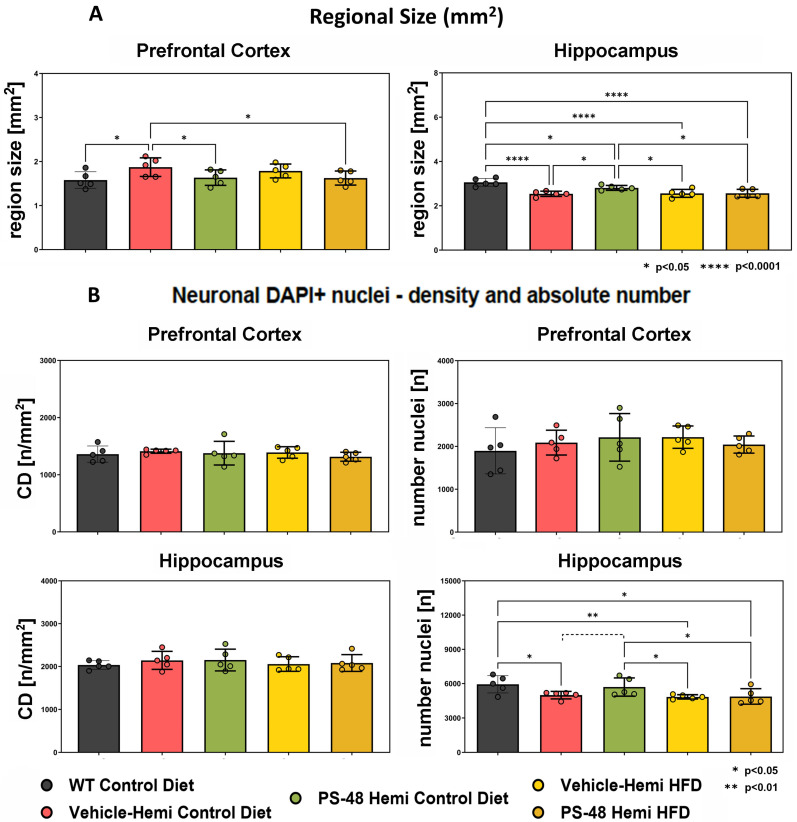
**Histology quantification.** (**A**) Region of interest (ROI) size. Unbiased delineation of prefrontal cortex (PFC) and hippocampus (HC) size is given as mm^2^ (average of 5 sections). The five animal groups correspond to wild type (WT) or transgenic hemizygote (TG); standard diet (SD) or high-fat diet (HFD); and whether diet-supplemented with vehicle (DMSO) or PS48. These are color coded according to the legend below. Compared to WT-CD, the TG-SD evidenced a larger mean PFC area (*p* < 0.05) that was reversed by PS48 (middle bar). A similar trend, not statistically significant (ns), is mirrored in the HFD fed mice (±1SE, n = 5). The HC, on the other hand, was considerably atrophied in the TG group compared to WT-SD under both standard and high-fat diet conditions (*p* < 0.001). PS48 reversed HC loss in the TG-SD group (middle bar, *p* < 0.05), whereas it did not do so in animals fed an HFD. (**B**) Neuronal nuclei density and regional number. Top row; there were no significant changes in density of neuronal-appearing, DAPI-positive nuclei (OD, left, given as n/mm^2^) or absolute number (n, right) in the PFC between groups. Bottom row: in the HC, there were similarly no changes in density between the 5 groups. However, in absolute number, TG on a SD had fewer total neuron-like nuclei than WT (*p* < 0.05), a result that PS48 partially reversed but without reaching statistical significance (middle bar, ----- *p* = 0.08). TG fed HFD with veh also had significantly lower total nuclei profiles than WT (*p* < 0.01), but the addition of PS48 proved ineffective for reversal. The HC regional size and absolute nuclei number results complement each other, consistent with an interpretation of neuro-degenerative cellular drop-out in TG mice and prevention by PS48, but this was effective only under standard diet conditions. * *p* < 0.05, ** *p* < 0.01, **** *p* < 0.0001.

We tested for anatomical evidence of neuroprotection and that PS48 reached its intended central target. Prefrontal and hippocampal regional sizes in sagittal sections taken from one brain hemisphere were measured (mm^2^). In the PFC, the TG animals showed a small increase in mean size, whereas the hippocampus revealed an opposite loss in volume relative to WT (*p* < 0.0001, n = 5 ea.) Figure 2A. Over both regions, PS48-treated mice on a SD saw a significant reversal of the transgene effect (*p* < 0.05). The TG animals on an HFD had just as severe hippocampal volume loss but did not respond to PS48. DAPI nuclei with neuronal characteristics were quantified by measuring optical density (OD, no./mm^2^, left graphs) and absolute number (n, right graphs) Figure 2B. The prefrontal cortex and hippocampus both showed relatively little change in nuclei density over the animal groups. However, in absolute numbers, the hippocampus sustained significant losses in TG mice on a SD (*p* < 0.05) or an HFD (*p* < 0.01) compared to WT. PS48 treatment again produced partial reversal under SD conditions, with the difference approaching but not reaching statistical significance (*p* = 0.08). There was no effect by this measure in the HFD group, unlike as was noted above, as seen in Figure 2B.

### 2.3. Immunohistochemistry: Phospho-Akt, -GSK-3β, and -Tau

Further evidence for target engagement was sought using quantitative immunofluorescence microscopy. Triple-labeled sections of PFC and hippocampus using antibodies vs. pAkt (S473), pGSK3β (S9), or pTau (T231) and β-amyloid and DAPI were examined under 10× magnification using the software-assisted adaptive threshold technique (see methods and Supplement Figure S2A,B). A further refinement was to quantify the signal profiles as either associated with amyloid plaques or as found in areas absent of plaques using a masking and digital subtraction format. The rationale was to investigate if spatial differences on an anatomic scale in the kinase activation pattern between β-amyloid plaque-containing and plaque-free areas could partially explain discrepancies found in the literature as to the phospho-status of Akt among the various reported AD models and disease severities. Protein studies on soluble vs. insoluble fractions have also sometimes resulted in contrasting results [29].

**Figure 3:**
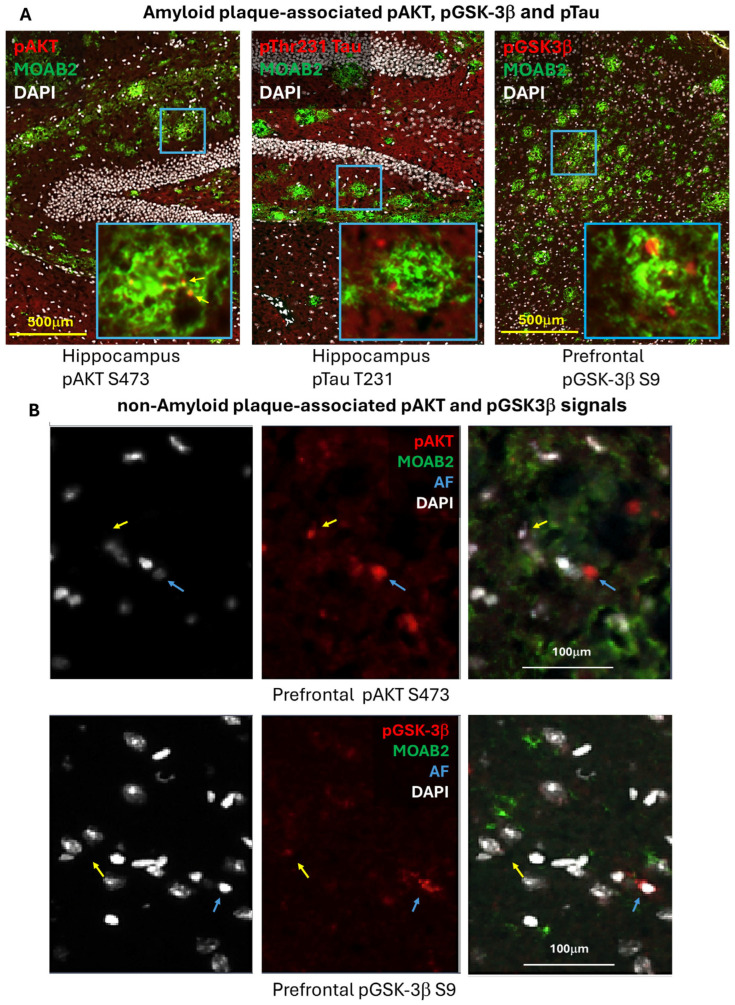
**Localization patterns of pAkt, pGSK3, and pTau immunofluorescence in TG mice.** (**A**) PhosphoS473 Akt, phosphoS9 GSK-3β, and phosphoT231 Tau signals were developed over both β-amyloid plaque-bearing and plaque-free regions, defined by co-localization with or absence of Aβ immunoreactivity (MOAB2 antibody, yellow). Left photomicrograph, representative pAKT is detected over an HC plaque (red signal indicated by fine arrows, insert); middle, pTau signal (red) is shown to be associated with an HC plaque; right, pGSK3β signal (red) over a PFC plaque. (**B**) Upper row: in non-plaque-containing areas of the PFC, a cellular pAkt signal (red, blue arrow, center panel) is distinguished from a non-specific autofluorescence signal (AF, yellow arrow, above it and composite fluorescence right panel) that was not associated with a nucleus signal (DAPI, left panel). Lower row: a pGSK3β signal (red, blue arrow, center panel) is clearly associated with a DAPI-defined nucleus (composite right panel). An autofluorescence signal (yellow arrow, above it and barely detectible in composite right panel) is also DAPI negative (left panel). All AF signals (green 488 nm) were filtered out in the quantification process. Scale = 100 μm.

**Figure 4:**
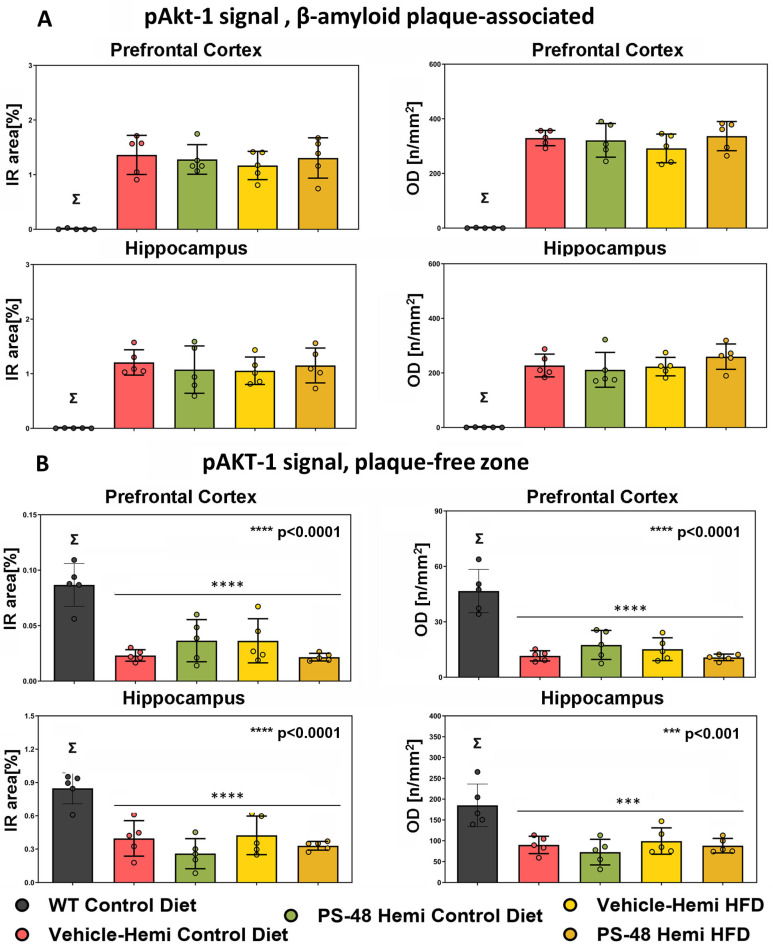
**Immunofluorescence signal quantification. pAkt.** (**A**) pAkt-1 signals over β-amyloid plaque areas in the PFC (upper row) and HC (lower row). Signal amounts are given as IR area (% of ROI size, left panel) and OD (optical density profiles, n/mm2, right panel). All transgenic groups had similar levels of pAkt. (Σ; in control WT animals, no plaques were detected). (**B**) pAkt-1 signals over plaque-free zones. Note the relative rarity of IR and OD signals by vertical axis scale as compared to plaque-associated Akt. In both the PFC and HC, control animals (absent any plaques) had the highest basal pAkt (activated) signals (Σ). All transgenic groups evidenced reduced (inhibited) pAkt signals relative to the WT control (**** *p* < 0.0001, *** *p* < 0.0005). Σ denotes mean (± standard error) of WT Control animals was significantly different from all other groups. WT Control data (Σ) registers zero in plaque-associated counts of (A) as such animals lacked β-amyloid deposits.

Examples of pAkt, pGSK, and pTau fluorescent signals associated with plaque are shown in Figure 3A. Examples of pAkt and pGSK profiles over non-plaque bearing areas in the PFC are shown in Figure 3B. Lipofuscin autofluorescence profiles were filtered out before quantification. In both examples, kinase signals are presumed to be cellular since they are associated with nuclei. The quantification of plaque-associated pAkt signals over the 4 TG mouse groups in the PFC and hippocampus did not show a change with respect to diet or treatment (IR: left, and OD: right variables, Figure 4A). However, in plaque-free zones of the PFC and hippocampus, TG animals showed an overall decrease in pAkt signal compared to WT (bar 1 vs. 2–5, *p* < 0.005, *p* < 0.05, respectively, Figure 4B). The TG/SD animals treated with PS48 showed only a modest reversal trend in the PFC (bar 3 vs. 2, ns), whereas no consistent trend in this measure was to be found in hippocampal sections.

**Figure 5:**
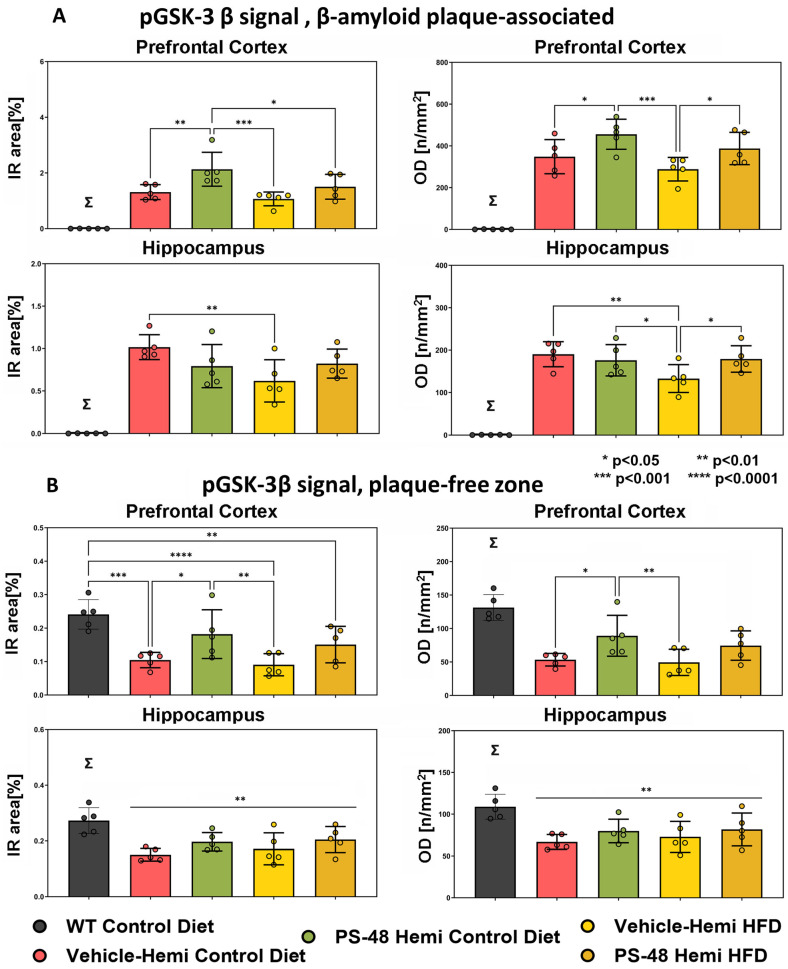
**Immunofluorescence signal quantification. pGSK-3β.** (**A**) pGSK-3β quantification over β-amyloid plaques. In PFC (top row), pGSK levels were increased (inhibited) in animals on a standard diet (SD) fed PS48 (middle vs. 2nd bar, *p* < 0.01, *p* < 0.05). In HFD fed mice, PS48 also trended (ns) or significantly (*p* < 0.05) increased levels of pGSK. In the HC (lower row), PS48 trended (ns) or significantly (*p* < 0.05) increased pGSK only in animals on an HFD (bar 5 vs. 4). HFD-alone (vehicle) animals showed reduced pGSK relative to SD/vehicle fed mice (bar 4 vs. 2, *p* < 0.01). (**B**) pGSK-3β quantification over Aβ plaque-free areas. pGSK levels were highest in both the PFC (upper row) and HC (lower row) control animals vs. all TG groups (** *p* < 0.01 and * *p* < 0.05, respectively), similar to the pAkt result above. TG mice on SD/vehicle showed significant reductions in non-plaque pGSK relative to WT (bar 2 vs. 1) in both the PFC and HC (*p* < 0.001). PS48 restored pGSK balance in the PFC (bar 3 vs. 2, *p* < 0.05), even to a level not significantly different from the control, by both metrics. In the HC, however, this was only a slight trend. Under HFD conditions in the PFC, PS48 only tended to restore pGSK levels in the direction of the control (bar 1) relative to the vehicle (bar 5 vs. 4, *p* = 0.07, ns) but was unable to do so in the HC. * *p* < 0.05, ** *p* < 0.01, **** *p* < 0.0001. Σ denotes mean of WT Control animals was significantly different from all other groups. WT Control data (Σ) registers zero in plaque-associated counts of (**A**) as such animals lacked β-amyloid deposits.

The pGSK3β results, as an endogenous proxy for Akt activity, proved more dramatic (Figure 5A,B). When pS9GSK-plaque overlapping profiles were quantified in the PFC using the two measures, the basal levels of pS9 (inhibited) GSK3-β in the TG/SD group were substantially increased by PS48 (bar 3 vs. 2, *p* < 0.01 and *p* < 0.05, upper row). TG animals on an HFD with reduced phosphorylation of GSK also benefited from PS48 (bar 5 vs. 4, upper row). While results in the hippocampus were less clear, TG/HFD animals showed similar recovery (*p* < 0.05, Figure 5A, lower row). In plaque-free zones of the PFC, where the signal in WT mice could be assessed, the results were just as clear, showing, for both the SD and HFD conditions, reduced pGSK in the vehicle (*p* < 0.0001) and partial reversal of GSK phosphorylation levels in PS48-treated mice (*p* < 0.05, upper row, Figure 5B). The data for the hippocampus in relatively plaque free areas did not reveal a significant treatment effect pattern aside from minor trends in the context of an overall expected reduction in pGSK signals across all TG mice groups (lower row).

**Figure 6:**
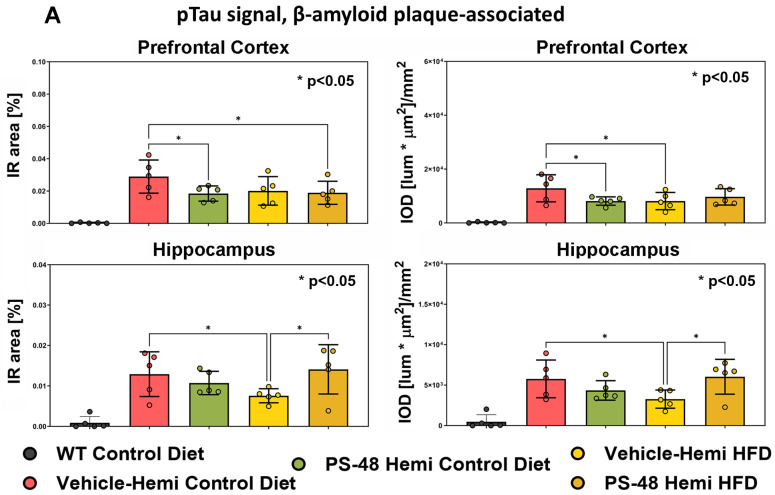

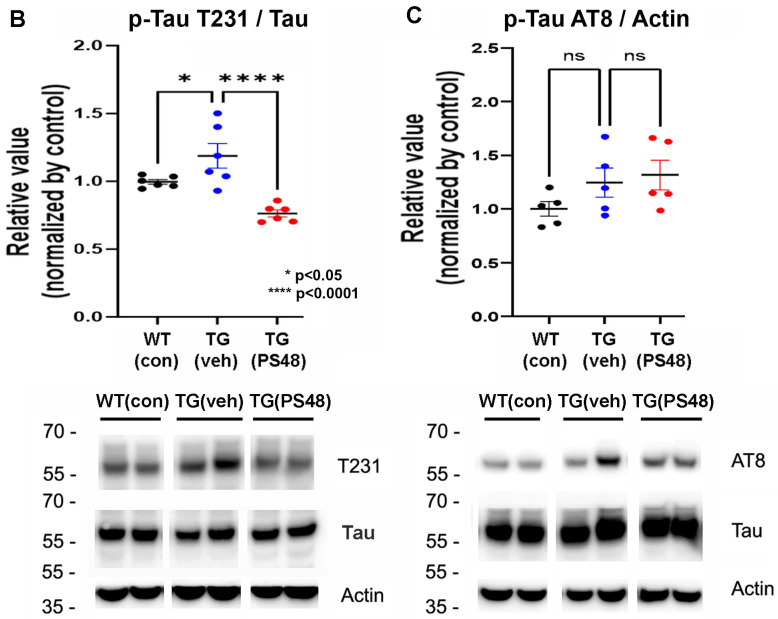
**pTau studies.** (**A**) Immunofluorescence quantification. pTauT231 signals were only found associated with Aβ-immunoreactive plaque laden areas in TG mice. In the PFC, PS48 significantly reduced pTau levels by both measures (IR and OD; bar 3 vs. 2, *p* < 0.05) in animals on a SD. However, HFD-fed animals did not benefit from PS48. In the HC, PS48 tended to decrease pTau levels under SD conditions (ns); however, under HFD conditions, a paradoxical increase in pTauT231 was obtained (bar 5 vs. 4, *p* < 0.05). (**B**) Western blot analysis of whole-brain lysates. Anti-phosphoTau T231 Western blot signals were corrected for total Tau and normalized to WT. TG mice had significantly higher pTau levels than WT (* *p* < 0.05). The addition of PS48 depressed pTauT231 levels to values below the control (*p* < 0.0001, n = 6 animals were quantified). Representative Western blots (n = 2) shown below, including actin loading control. (**C**) PhosphoTau epitope AT8 (pS202/pT205) signals tended to be higher in TG mice relative to WT (not significant, ns). PS48 did not affect anti AT8-detected pTau (normalized to actin, n = 5 were quantified). Representative Western blots shown below (n = 2). * *p* < 0.05, **** *p* < 0.0001.

Given the more robust immunohistochemical results showing GSK3-β activation (low pS9 GSK) in TG animals and evidence for partial reversal by PS48, compared to the corresponding pAkt changes, we tested for pTauT231 which has predicted sensitivity to GSK3-β activity. Anti-pTau fluorescence signals (immunoreactive surface area percentage (IR area) and relative integrated optical density (IOD) variables) were quantified around plaque-associated dystrophic neurites. In Figure 6A, increased pTau is found across all TG groups. Consistent with the pGSK3-β results, the pTau burden is significantly lessened by PS48 in the PFC of SD-fed mice (*p* < 0.05). It is not clear, however, why, in the TG group on an HFD (PFC), the pTau levels were uniformly low (bars 4 and 5). In the hippocampus of TG mice (Figure 6A, lower row), pTau was also found across all groups, and again, PS48 tended to reduce pTau in SD-fed animals but to a degree not reaching significance. In the hippocampus, pTau results in the TG/HFD group were again surprisingly reduced and even paradoxically brought up to TG/SD levels by PS48. With the positive pTauT231 results noted in PS48-treated animals on a SD, at least in the PFC, we tested pTau using a straight Western blot, focusing on the hippocampus of those animals fed AN SD (Figure 6B). Using this measure, it is shown that the transgene elevated pTauT231 levels relative to WT (*p* < 0.05) and that PS48 knocked it down below the WT baseline (*p* < 0.001) (summary ANOVA; F(2,12) = 14.79, *p* = 0.0001). The phospho-AT8 epitope (S202/T205), having a lessened GSK3-β preference for a Cyclin-dependent kinase 5 (Cdk5) priming phosphorylation reaction compared to AT180 (T231) [45], was also increased in TG animals but did not respond to PS48 (Figure 6C) (ANOVA; F(2,12) = 1.97, *p* = 0.18, ns).

### 2.4. β-Amyloid and PS48 Level Quantification

We lastly determined levels of β-amyloid (Aβ) and PS48 in the prefrontal cerebral hemisphere (PFC) and cerebellum, respectively, to confirm transgene expression and drug delivery. Aβ peptides were undetectable by ELISA in wild-type brains, whereas high levels of Aβ species ranked Aβ42 > Aβ40 > Aβ38 were present in all TG animals. Neither diet nor PS48 treatment had any effect on Aβ42,40 levels. Aβ38 was significantly elevated in TG groups (no. 4 and 5) on an HFD (Supplement Figure S3A). Tandem MS data on cerebellar tissue confirmed high PS48 levels in those groups that were accordingly treated, whereas ions remained barely detectable in groups no.1,2,4 that were not exposed (*p* < 0.0001) (Supplement Figure S3B). TG animals fed an HFD with PS48 had higher levels than treated TG animals on a SD (group 5 vs. 3, *p* < 0.005). A sample MS spectrum is shown in Supplement Figure S3C. β amyloid plaque quantification by immunofluorescence (MOAB-2, a pan-Aβ antibody) also confirmed transgene expression status in groups 2–5 (Supplement Figure S3D). Similar to ELISA results, there was no change in plaque levels with either diet or treatment intervention.

## 3. Discussion

The outcomes of most of the presented experiments support our conclusion that oral ingestion of PS48 reached its molecular target, the PI3K-PDK-1/Akt-1 activation axis, in the APPswe/PS1E9 AD transgenic mouse brain on either diet regimen. PS48 is a small-molecule (MW 286.8) chlorophenylpentenoic acid that binds to the HM (PIF) pocket of PDK-1 in its regulatory N’ lobe, thereby enabling a stabilization and allosteric activation of Akt bound to its substrate docking site [40,46,47,48,49].

Significant reversals of the transgene biochemical phenotype, i.e., inhibited basal Akt activation status (Figure 1B, high-fat diet—HFD; Supplement Figure S1C, -HFD) and Akt enzymatic activity (Figure 1C, HFD) along with downstream overactivation of GSK3β (Figure 5A,B, SD and HFD) and hyperphosphorylation of Tau at T231(Figure 6B, SD), by PS48 are shown. Under standard diet—SD—conditions, the improvement from basal levels with PS48 was a trend that did not quite reach statistical significance (Figure 1A) but did adjust to a level that was not significantly different from wild-type—WT—control. Clearer results tended to be obtained when Akt was first immunoprecipitated before Western blot or measurement of its activity, as compared to straight Western blots that fractionate crude whole-brain lysates. For instance, crude lysates from TG animals on a SD showed only a small trend in pAkt/totAkt ratio change, still favoring PS48 (Supplement Figure S1A), whereas the difference was more noticeable when first immunoprecipitated (Figure 1A). Similarly, under HFD conditions, immunoprecipitated Akt activity shows more significant restoration by PS48 (Figure 1C) than that found in crude fractions (pAkt, Supplement Figure S1C). The same trend, albeit not reaching statistical significance, can be said of endogenous pGSK in those crude lysates (Supplement Figure S1B,C). Additionally, occasional experiments did not show the expected reduction in the pAkt/totAkt ratio in TG animals on an HFD that were fed the vehicle (Figure 1B and Supplement Figure S1C), but their outcome still favored PS48. The reason behind some of these inconsistencies is not clear but variance from inter-animal diet x disease interactions and the vagaries of kinase quantification in brain samples can be addressed with larger group sizes in our future work.

In measures of neuronal attrition, i.e., regional size and neuron number, PS48 partly but significantly reversed hippocampal loss in TG animals on SD, but not on HFD (Figure 2A,B). This would be consistent with its hippocampal neurogenesis effect [50], as well as more general trophic effects of PI3K/Akt activation on hippocampal synaptogenesis [51]. Interestingly, in the PFC, there was a significant but opposite increase in region size in TG animals on a SD along with a trend toward higher absolute neuronal number. PS48 normalized the increase too. It is intriguing to speculate that the trophic PFC phenomenon may correspond to a compensatory increase in the executive control network, as noted in AD resting state functional connectivity fMRI studies [52,53].

To better understand brain region and local Aβ-dependent effects on the state of Akt activation, we parsed activated Akt-pS473 and inhibited GSK3β-pS9 immunohistology into Aβ plaque-laden and plaque-free areas within the PFC and hippocampus. The most significant findings were in the plaque-free zones of both regions, where the transgene effect under either SD or HFD conditions was to activate (under phosphorylate) GSK3β. PS48 rescued pGSK3β to inactivation levels in the PFC but only trended towards doing so in the hippocampus (Figure 5B). In plaque-affected areas, PS48 again drove S9GSK phosphorylation in the PFC under both diet conditions (Figure 5A) but proved less effective in the hippocampus, albeit working better there in HFD-fed animals. The direction of pGSK change in these immunohistological studies is consistent with Figure 1 Western blot data and, therefore, is logically ascribed to the linked inhibition (transgene effect) and restoration (PS48 effect) of its regulatory kinase, namely Akt. Turning to Akt itself, we saw no change in pAkt with PS48 in Aβ plaque-affected zones. In plaque-free zones of the PFC and hippocampus, while basal pAkt was significantly inhibited in TG mice, as expected, PS48 had no effect save for a positive trend in the PFC of animals fed an SD. The reasons for this may be technical, noting first a relative paucity of pAkt signal profiles in areas unaffected by Aβ in the transgenics but also the obvious differences in detection method and secondary antibodies used in fluorescence histology as compared to Western fractionation. It could also be attributed to differential brain region drug availability. In summary, the changes in pGSK (plaque-free or plaque-associated) and pAkt (plaque-free) immunostaining in the presence of PS48 were generally more evident in the PFC than the hippocampus. The Western/IP, Akt, and GSK data, coming only from hippocampus/diencephalon dissections, nevertheless showed consistent transgene and drug effect results in this brain region.

PS48 significantly reduced Tau phosphorylation at T231 (AT180) in brain homogenates (Figure 6B) and by immuno-fluorescence histology (IFH) (Figure 6A) in the PFC of TG mice on SD. A trend in the same direction of change is confirmed by IFH in the hippocampus. The link binding Tau hyperphosphorylation to PI3K/Akt inhibition via GSK3β disinhibition is experimentally well documented [54,55]. In this study, we did not additionally test the purported direct phosphorylation of Tau by Akt on T212 and S214 [56]. Hyperphospho-T231, a marker of dystrophic neurites associated with plaques, has not been previously investigated in this mouse model, with or without HFD, as far as we are aware. Two other phospho-epitopes, AT8 (S202/T205, Figure 6C) and PHF-1 (S396) did not respond to PS48. The exaggerated phosphorylation status of these sites has been reported on in this model [57,58]. One technical explanation for the preferential PS48 effect is that the observed increase in phosphorylations of the latter two sites in the TG mice fed the vehicle was not as significant as it was for T231, therefore making any change less obvious. It may also be related to the differential specificity of GSK3β, a major proline-directed Tau kinase, for T231. For instance, while all three sites can be independently phosphorylated by GSK3β (and cdk5 [59]), they are primed to different degrees by other kinases (e.g., cdk5, CAMKll, and PKA) [60]. T231 phosphorylation may be more priming dependent, while AT8 and PHF-1 sites are relatively unprimed [61]. The T231 and S202/T205 or S396 sites are further differentiated by the former having a more critical role in normal microtubule binding [62,63] and is hyperphosphorylated at an earlier Braak stage in disease progression [64,65]. Finally, the differential effect of PS48 on Tau epitope phosphorylation levels could also be species-brain-substrate- and/or drug-concentration-dependent. For instance, in a preliminary experiment using neuronal organoid cultures made from IPSCs obtained from an AD patient, phospho-AT8 Tau levels in lysates were reduced by ~50% after a 4-day application of 10 μM PS48.

For unclear reasons, PS48 did not impact pTau under HFD conditions, and this was especially the case in the hippocampus (Figure 6A and in Western blots) In a possibly related manner, hippocampal regional atrophy also did not respond to PS48 in the same animals raised on an HFD (Figure 2B). Thus, the more substantial improvement in cognition we reported in these HFD-induced pre-diabetic and overweight transgenic mice due to PS48 treatment [44] was not reflected here by the expected hippocampal neuroprotection, whereas it was reflected in animals raised on a SD. Nonetheless, PS48 restored Akt and GSK phosphorylations in the hippocampus of HFD-fed mice as shown in Figure 1B,C and Figure 5A. One possibility for the curious disparity in hippocampal neuroprotection by PS48 is that TG animals on an HFD had greater vasculopathy and/or inflammation burden [57,58], rendering Tau phosphorylation and neuronal loss less responsive to drugs. Neither pathology was tested for in this work. However, due to PS48’s other actions to reduce weight and improve glycemic control that we reported, this discrepancy would not predict outright that PS48 would have less efficacy in AD patients who are also diabetic.

As expected from its properties, PS48 did not affect Aβ 42 and 40 levels between the groups. It is interesting that Aβ38 levels were significantly higher in the TG on an HFD group but were also accentuated by PS48. Aβ 38 is shown to negatively regulate the more aggregate prone Aβ42 species and is associated with lessened AD pathology [66,67]. Another interesting finding is that PS48 levels were higher in the transgenic HFD brains compared to the SD brains. The possibility of an HFD compromising BBB permeability and function to affect drug delivery [68,69] will be addressed in a future pharmacokinetic study.

Other chemicals and herbals that activate Akt and tamp down activated GSK3β have also been found to improve spatial memory and increase neuronal survival or reduce phospho-Tau in AD transgenic mice and other models. They do not appear to over-activate Akt in wild-type backgrounds. Examples include the following: pyrrolidine dithiocarbamate [70], SC79 [71,72], salidroside [73], Tanshinone [74], Fasudil [75], curcumin [76], and Leptin [77]. The majority of our data do not predict that chronic PS48 treatment will lead to constitutive over-activation of Akt and heighten the theoretical risk of oncogenesis. Most carcinomas associated with PI3K/Akt dysregulation, commonly in tissues also overexpressing hormone receptors, are from the loss of the PTEN suppressor function or mutation of PI3K. Our experiments were limited to the brain and tend to show just a partial correction of an AD-related under-activation of Akt. Moreover, PS48 is not a direct agonist. Although our mice lived to 14 months without obvious systemic tumorigenesis on necropsy or weight loss, there was mild biochemical liver dysfunction. Body organs with a high metabolic reprogramming risk, such as the liver, could conceivably be susceptible to PI3K/Akt pathway stimulation, even resulting in hepatocellular carcinoma. We plan to fully address these potential concerns in a future pharmacokinetic ADMET study.

Based on our results and those reported on the other compounds above, there has been a call for AD treatment trials that target Akt homeostasis [78,79,80]. Another property of PS48 aside from hippocampal neurogenesis is that it may stimulate the Warburg effect or aerobic glycolysis, having an anti-apoptotic effect but not necessarily being associated with cell proliferation [81,82]. To date, the only drugs with disease modifying potential approved for clinical use in MCI and early AD are the monoclonal anti-Aβ antibodies, Lecanemab and Donanemab [83,84]. There are concerns over their effectiveness, risk of ARIA inflammatory and vascular side effects, contraindications, and global availability, as well as cost [85]. As clinical trials with the monoclonals have shown, the APOE4 gene dosage is critical to therapeutic efficacy and side effects. Moreover, since carrying an ApoE4 allele negatively affected therapeutic responses in early intranasal insulin trials [86], future clinical testing of insulin pathway modulating drugs, such as PS48, certain analogs [43], and other allosteric activators [87], should stratify side effects and benefit metrics according to APOE genotype. The search should continue for effective, orally administered, small molecules that target a critically deficient signal pathway in AD, have an acceptable safety profile, and are relatively inexpensive to formulate.

## 4. Methods

### 4.1. Animals and Oral Drug Dosing

Details of the wild-type (WT) and transgenic (TG) mice used in the present study of drug–brain targeting have been published, including IACUC approval (approval code no. CMTT# 0118-10, 2 July 2014) [44]. Briefly, double AD mutant (APPswe and PSEN1dE9) transgenic mice (AZ B6C3-Tg 85 Dbo/MmJax stock 004462) were supplied by Jackson Labs (Bar Harbor, ME, USA) [88,89]. All mice were male. WT littermates were used as controls. At ~2 months age (9 weeks), when plaques are first deposited, they were randomly assigned to standard (SD) or high-fat (HFD) diets and at 10 months of age (44 weeks) were transitioned to daily SD or HFD supplemented or infused with the study drug (PS48) or vehicle (V) for oral ingestion. This resulted in 5 animal groups (1. WT-SD, 2. TG-SD-V, 3. TG-SD-PS48, 4. TG-HFD-V, and 5. TG-HFD-PS48). Each group was initially populated with 10 animals; after attrition, 36 animals remained, with 24 in groups 1, 2, and 3 and 12 in groups 4 and 5. From 12 until 14 months of age, cognitive testing was carried out, shortly after which the animals were euthanized and saline-perfused (see Table 1). Hemibrains were harvested; one side was embedded in OCT for immunohistochemistry, the other flash frozen in liquid nitrogen for protein assays and stored at −80 °C. The cerebellum was isolated and flash frozen for tandem mass spectrometry.

Rodent diets were obtained from Research Diets Inc. (www.researchdiets.com; New Brunswick, NJ, USA. URL accessed on 5 April 2024): standard (control) diet (SD); #D1245OH1 is 10% Kcal fat. High-fat diet (HFD); #D124511 is 45% Kcal fat. Transgenic cookie dough was purchased from Bio-Serve Inc. (www.bio-serve.com/CTUS; Flemington, NJ, USA. URL accessed on 5 April 2024); #53472 is 12.5% kcal fat.

PS48 (MW. 286.75), Z enantiomer, was bulk synthesized in 10 g lots of dry powder according to Stroba et al., 2009 [46] and purified through 2 rounds of re-crystallization to 99% as confirmed by ^1^H-NMR and high-resolution LC mass spectrometry.

Mice were orally dosed with 50 mg PS48/Kg/day. Based on a mean adult transgenic mouse weight of 46 g, the daily dose of PS48 was 2.3 mg. The duration of drug therapy from the outset through harvesting was just over 4 months.

Antibodies and reagents for Western blot and IP:

Anti-Akt-1 (total), mouse IgG mAb B-1 (Santa Cruz Biotechnology, (SCBT), Dallas, TX, USA) Sc-5298, 1:1000 for Western blot and 1:100 for IP); anti-phospho-Ser473Akt, rabbit mAb D9E (Cell Signaling, Danvers, MA, USA) no. 4060 1:1000) or anti-Akt-1/Akt-pS473 (PhosphoPlus DUET, Cell Signaling, Danvers, MA, USA) no. 8200). Anti-GSK-3α/β (total) rabbit IgG mAb D75D3 (Cell Signaling, no. 5676, 1:1000), anti-phospho-Ser21/9 GSK-3α/β (Cell Signaling, no. 5558 1:1000) or anti GSK/pGSKS9 rabbit mAb IgG (PhosphoPlus DUET, Cell Signaling, no. 8213), both against the 50 kDa endogenous GSK-3α/β. Anti-Actin (Cell Signaling, no.3700, 1:1000). Anti-Tau (total), rabbit mAb IgG, D1M9X (Cell Signaling, no. 46687, 1:1000), anti-phosphoTau Ser396, mouse mAb IgG, PHF13 (Cell Signaling, no. 9632, 1:1000) or anti-Tau/pTauS396 (PhosphoPlus DUET, Cell Signaling, no.56285); anti-phosphoTau Thr231 (Cell Signaling, no. 71429, 1:1000); anti-phosphoTau Ser202/Thr205, AT8, mouse mAb (Invitrogen, Carlsbad, CA, USA) no. Mn1020, 1:1000). Anti-rabbit IgG-HRP linked (Cell Signaling, no. 7074). GSK-3β fusion peptide (Cell Signaling, no. 9237, crosstide-paramyosin, a 27 kDa substrate for Akt phosphorylation).

### 4.2. Brain Lysates

PhosphoSafe Extraction reagent (EMD-Millipore, Burlington, MA, USA) and lysing Matrix D beads (MP Biomedicals, Solon, OH, USA) were added to an Eppendorf tube with the brain samples. The tissue was disrupted using a FastPrep-24 homogenizer (MP Biomedicals) at high speed (e.g., 6.5 m/s) and for short duration (2 × 30 s). After homogenization, the samples were centrifuged at a high speed (14,000 rpm) for 10 min at 4 °C. The soluble lysate was transferred to the new tube and stored at −80 °C for long-term storage until use. Protein concentrations ranged from 7.70 to 9.25 mg/mL.

### 4.3. Western Blot Analysis

Whole-brain tissue extracts were used for direct Western blot analysis. These were prepared from hippocampal/temporal/diencephalon samples dissected from frozen hemibrain and homogenized in lysis buffer. Protein concentrations were determined, and volumes were adjusted for normalization. Then, 20–30 μg, depending on comb size, was diluted into Laemmli sample buffer, heated (95 °C, 10 min), cleared by centrifugation, and separated on SDS–PAGE. After transferring to PVDF membranes (Immobilon-P; MilliporeSigma, Burlington, MA, USA), the membranes were blocked in TBS containing 0.3% Tween-20 and 5% (wt/vol) non-fat dry milk. After incubation with primary antibodies (18 h at 4 °C in buffer containing 5% BSA and 0.05% NaN_3_), blots were washed and incubated in HRP-conjugated secondary antibodies (1:2000 dilution; Cell Signaling). Signals were detected using ECL reagents and quantified using a Kodak Image Station 4000R (Molecular Bioimaging, Bend, OR, USA). 

### 4.4. Akt1 Activity Assay

Akt1 was immunoprecipitated (IP) overnight from 200 μg of wild-type or transgenic mice brain extracts by first adding 2 μg (10 μL) of goat anti-Akt1 antibody. The following morning, 20 μL of 50% slurry of protein A/G-agarose (SCBT) was added for 1.5 h. After low-speed centrifugation, the collected beads were washed twice in kinase buffer (25 mM Tris-HCl (pH 7.5), 5 mM beta-glycerophosphate, 2 mM dithiothreitol (DTT), 0.1 mM Na3VO4, 10 mM MgCl_2_). Next, GSK-3-paramyosin fusion protein (crosstide, 1 μg/50 μL, 1.0 μg) was added in the presence of kinase buffer and 200 μM ATP, and the reaction (50 μL) was incubated for 30 min at 30 °C. The reaction was stopped by adding 50 μL of 2× sample Laemmli buffer. A sample thereof (10 μL) was loaded onto a 4–12% polyacrylamide bis-tris gel (Invitrogen) for fractionation.

### 4.5. Histology and Immunofluorescence

Brain hemispheres were imbedded in freezing medium, OCT, and dry ice-cooled with liquid isopentane into cryomolds. These were sagittal sectioned on a Leica CM 1950 cryotome (Leica Biosystems, Deer Park, Illinois, USA) at 10-micron thickness. Once the region of interest (ROI) was reached, 5 consecutive sections were kept per level, discarding the next 25, until 12 levels were collected in each of prefrontal cortex and hippocampus.

Systematic random sets of 5 levels having 5 sections each were labelled for phospho-Akt S473 (Rabbit, Cell Signaling, no. 4060S), pan β-Amyloid (Mouse, MOAB-2, Abcam, (Cambridge, United Kingdom) no. ab126649), phospho-GSK-3β S9 (Rabbit, Cell Signaling, no. 5558S), and phospho-Tau T231 (Rabbit, Abcam, no. ab151559). Secondary antibodies: Donkey anti- Rabbit Alexa fluor 647 (Abcam, no. ab150075) and Donkey anti-Mouse Alexa Fluor 488 (Abcam, no. A-21202). Each section was double labeled with MOAB-2 vs. amyloid to quantify signals in both plaque- and non-plaque-containing regions and counterstained with DAPI to identify nuclei and their heterogeneity characteristics. Non-specific binding to endogenous mouse IgG was blocked with M.O.M. serum (Vector Laboratories, Newark, CA, USA) no. MKB 2213-1) before primary incubation. Standard negative controls included the absence of primary antibodies. Sections were imaged at 10× on a Zeiss AxioScan.Z1 (Oberkochen, Germany) microscope equipped with LED illumination and band pass filter sets corresponding to each fluorophore. Images were captured with an ORCA Flash 4.0 B&W camera. The program software used for image analysis was Image Pro Premier v9 (Media Cybernetics Inc., Rockville, MD, USA).

### 4.6. Quantification

Brain regional (ROI, i.e., hippocampus and PFC) sizes were determined using delineation based on the Allen Institute Reference Brain Atlas (http://atlas.brain-map.org URL accessed on 5 April 2024) mouse sagittal section. The following unbiased variables were collected for all protein markers:

1. Mean signal (lumens);
2. Immunoreactive surface area (IR, noted as % of ROI size);
3. Object density (OD, noted as number of objects per mm^2^);
4. Relative integrated optical density (IOD, noted as lumen × μm^2^/mm^2^).

Histogram-based, limited adaptive thresholds were used to filter out captured objects below a size range (20–50 μm^2^) and the 8-bit grey value (0–255) limit. The calculated threshold varied per measurement of a particular antibody or DAPI-stained section based on the mean signal and standard deviation as well as an assigned sensitivity factor. Cell densities were evaluated by requiring a DAPI nucleus to have a minimal size of 20 μm^2^, meaning a full nucleated cell was present within the section. Moreover, for determination of neuronal (only) signals, neuronal nuclei were directly distinguished from glial-type cells based on a heterogeneity parameter (fraction of pixels that deviate more than the average intensity by >10%—its limit was set at 0.6 for separating neuronal nuclei (more homogeneous) from those of glial origin (higher heterogeneity)). pAkt, pGSK-3β, and pTau signals that were amyloid plaque-associated, i.e. overlapping with Aβ deposits, were segmented using an inverted plaque mask, derived from the MOAB-2 stain pattern, that was subtracted from the original (e.g., pAkt) to veil or set to zero any signals other than those related to plaques. Lipofuscin autofluorescence was similarly filtered using autofluorescence in the green (unlabeled) emission channel.

### 4.7. β-Amyloid Levels (ELISA)

Quantitative analysis for levels of Aβ 38, 40, and 42 were carried out on the anterior cerebral cortex, including the frontal cortex, corpus callosum, and basal forebrain (60–75 mg) but excluding the olfactory bulb. These were dissected from the flash-frozen hemi-brain samples rostral to the hippocampus and homogenized according to instructions in the Mesoscale Discovery (MSD) Aβ multiplex and V-plex mouse kits (Rockville, MD, USA; www.mesoscale.com, URL accessed on 5 April 2024). The samples were broken by a pestle in liquid nitrogen and transferred to an Eppendorf tube to which 5× eq. (*v*/*w*) of 5 M Guanadine HCl in 50 mM Tris pH 8.0 was added. The contents were homogenized by TissueLyserLT (Qiagen Sciences, Inc., Germantown, MD, USA) at a speed of 50. After centrifugation at 16,000× *g* for 20 min at 4°, the supernatants (300–400 μL) were sampled for protein concentration by a BCA assay for subsequent normalization adjustment (final 2.0 mg/mL). The lysate was diluted 1:10 in MSD Diluent35 and 25 μL were added to each well. The detection antibody was conjugated, namely 4G8. The total 4-spot well volume was 50 μL. Calibrations were carried out according to instructions using the supplied Aβ peptides and 7 serial dilutions ranging from 0.5 to 1000 pg/mL. Aβ levels were read out as pg/mL reflecting final concentrations after all dilutions according to the fitted calibration curves. All samples were measured in duplicate.

### 4.8. PS48 Levels (MS-MS)

Cerebellum specimens (~100 mg) were weighed and homogenized (Omni beadrupter) in extraction solvent (80:20 H_2_O/acetonitrile) and adjusted to 4× eq. (*w*/*v*). After a 20× dilution in 10 ng/mL Glyburide (as an internal standard)/acetonitrile using a 96-well format and centrifugation at 4000 rpm, 5 μL was injected into a Shimadzu LC-40 HPLC pump (Shimadzu Scientific Instruments, Columbia, MD, USA), Sciex 7500 electrospray LC tandem MS system (Sciex, Framingham, MA, USA). Eleven PS48 (MW 285.1) standard dilutions (0.5 to 10,000 ng/mL) were used to construct a calibration curve from clear peak intensities at 1.015 to 1.024 min. Calculated PS48 out concentrations are reported in ng/gm brain weight.

### 4.9. Statistics

Histological data were compared using 1-way ANOVA and Fishers Least Significant Difference (LSD) post hoc testing. Western blot densitometry data underwent 1-way ANOVA with post hoc analysis between groups using Tukey’s (HSD) multiple comparison testing, GraphPadPrism v10 software package.

## 5. Conclusions

PS48 is a small-molecule, allosteric positive modulator of PDK-1, an essential kinase in the activation sequence of PI3K/Akt kinase in response to insulin. The compound has the ability to alleviate one of several causes of insulin resistance in Alzheimer’s disease models, both in vitro and in vivo, as developed here. It does not appear to significantly overstimulate Akt activity in instances where the kinase is baseline inhibited, rather providing a correction. Oral-administered PS48 has beneficial action on cognition in transgenic mice and in the same mice is shown here to derive from access to relevant brain regions and expected molecular targets. In addition, evidence suggests improvement in hippocampal cell and volume losses as well as in pTau accumulation.

## Figures and Tables

**Table 1:** Animal Groups and Study Design.

Group no.	Group Label (Genotype-Diet-Drug)	Final n	Diet Intervention at 2 mo.	Oral Drug or Vehicle at 10 mo.	Behavioral Testing 12 to 14 mo.	Study Terminated 14 mo. n for Westerns
1	WT-SD	8	standard (SD)	-		5
2	TG-SD-V	8	standard (SD)	vehicle (V)		5
3	TG-SD-PS48	8	standard (SD)	PS48		5
4	TG-HFD-V	6	high fat (HFD)	vehicle (V)		5
5	TG-HFD-PS48	6	high fat (HFD)	PS48		5

## Data Availability

The data sets used or analyzed during this study are available from the corresponding author on reasonable request. Data sets generated from fluorescence and Western blot studies are additionally included in the Supplementary Information files.

## Supplementary Materials

The following supporting information can be downloaded at: https://www.mdpi.com/article/10.3390/ijms26083473/s1.

## Abbreviations

T2DM (type 2 Diabetes Mellitus); IR (insulin resistance); MWM (Morris Water Maze); SD (standard diet); HFD (high fat diet); OGTT (oral glucose tolerance test); AD (Alzheimer’s Disease); Akt (PKB, protein kinase B), PDK-1 (phosphoinositide-dependent kinase), mTOR (mechanistic Target of Rapamycin), GSK-3β (glycogen synthetase kinase), APOE (apolipoprotein E), pTau (phosphorylated Tau), Aβ (beta-amyloid peptide), PFC (prefrontal cortex), IP (immunoprecipitation).
