# Supplementary material for: Target Validation Studies of PS48, a PDK-1 Allosteric Agonist, for the Treatment of Alzheimer’s Disease Phenotype in APP/PS1 Transgenic Mice
Source: Int J Mol Sci. 2025 Apr 8;26(8):3473. doi: 10.3390/ijms26083473 (PMC12027031; doi:10.3390/ijms26083473)
Supplement: Supplementary file 1 [file ijms-26-03473-s001.zip › ijms-3491137-supplementary.pdf]

International Journal of *Molecular Sciences*

Article

Target Validation Studies of PS48, a PDK-1 Allosteric Agonist, for the Treatment of Alzheimer's Disease Phenotype in APP/PS1 Transgenic Mice

Henry W Querfurth [1]*, Cynthia Lemere [2], Jason Ciola [2], Daniel Havas [3], Weiming Xia [4] and Han Kyu Lee [5]

1 Tufts Medical Center, Dept of Neurology and Tufts University School of Medicine, Dept of Neuroscience 800 Washington St, Boston, MA 02111, United States <hquerfurth@tuftsmedicalcenter.org>
2 Brigham and Women's Hospital, ARCND, 60 Fenwood Rd. Hale Bldg. for Transformative Medicine, Boston MA 02115, United States <clemere@bwh.harvard.edu>, <Jason_christopher.ciola@mailbox.tu-dresden.de>
3 Psychogenics Inc. 215 College Rd. Paramus, NJ 07652, United States <daniel.havas@psychogenics.com>
4 Boston University, Chobanian and Avedisian School of Medicine, Dept. of Pharmacology, Physiology and Biophysics 72 E. Concord St. Boston, MA 02118, United States <wxia@bu.edu>
5 Tufts Medical Center, Dept. of Neurology, 800 Washington St, Boston, MA 02111, United States <hlee@tuftsmedicine.org>
* Correspondence: hquerfurth@tuftsmedicalcenter.org

Supplementary Material Figure Legends

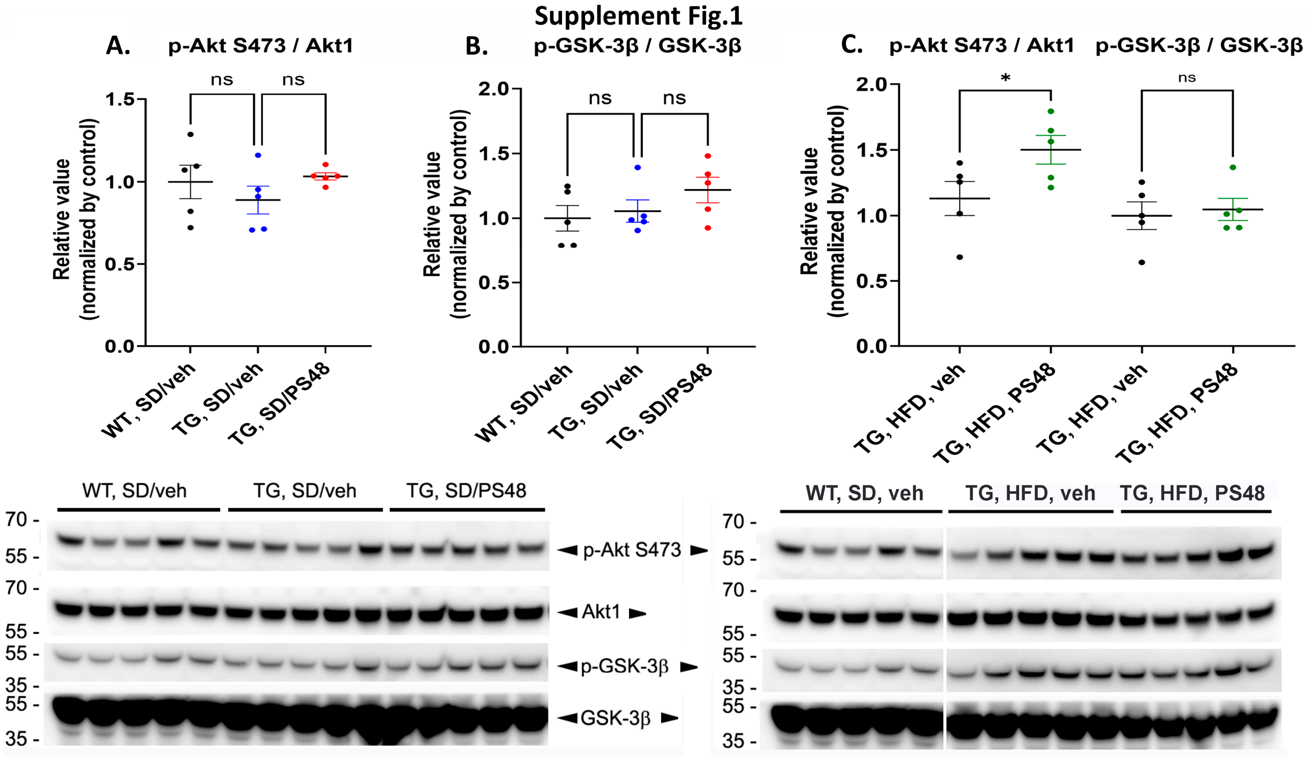

Figure S1. Whole tissue lysate; straight Westerns of endogenous Akt and GSK3β. (A). Left panel. Whole tissue lysates from hemi brain section containing HC/temporal cortex/thalamus of TG mice raised on SD/vehicle showed a slight depression of the pAkt/ totalAkt ratio compared to WT animals (ns). TG animals fed PS48 for 6 months, rebounded to a small degree (ns). Representative Western shown below (n=5). pAkt-S473 corrected for total Akt then normalized to WT. (**B**). Middle panel. Similarly, endogenous GSK-3β/ total GSK ratio showed a small increment in TG animals fed PS48-supplemented SD compared to WT control (ns, p=0.15) and TG/ vehicle (ns). The trends suggest a functional PS48-mediated enhancement of PAkt in SD fed animals. (**C**). Right panel. In a separate analysis of animals raised on HFD compared to

 https://doi.org/10.3390/ijms26083473

the same WT control group on SD as above, TG-HFD mice fed PS48 showed a significant increase in pAkt/ totAkt ratio ($p<0.05$) compared to vehicle (ANOVA, $F(2,12)=6.67$, $p=0.01$). However, the same HFD animals showed no significant change (ns) in endogenous pGSK/ total GSK normalized ratio when fed PS48. Representative straight Western is below.

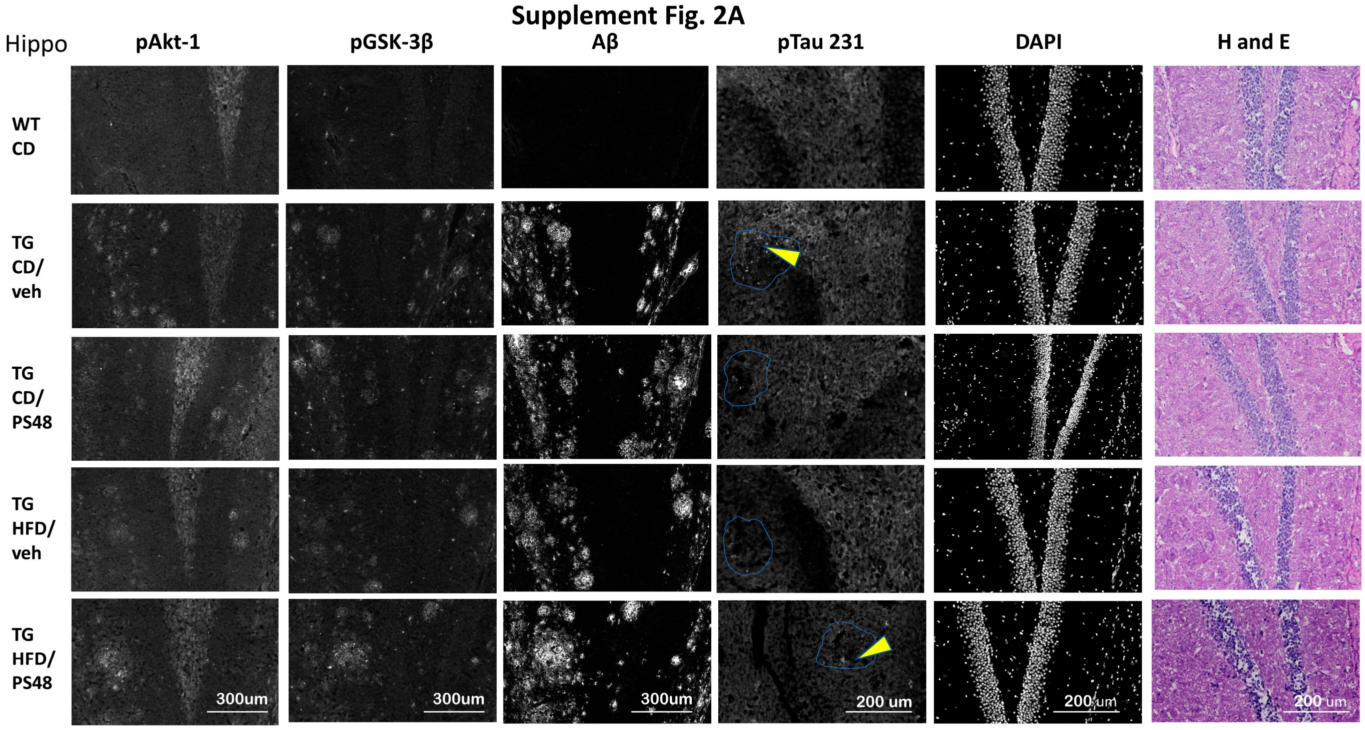

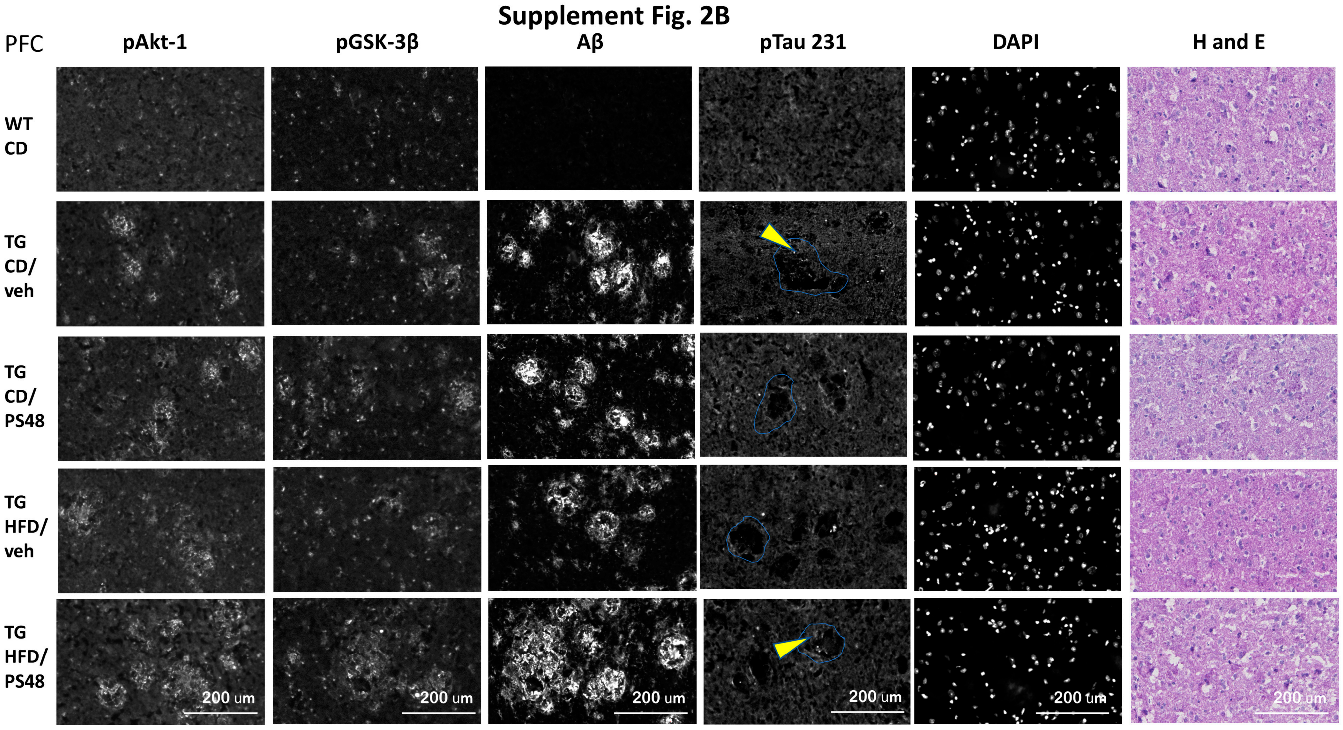

Figure S2. Immunofluorescence labeling of Hippocampus (A). and Prefrontal Cortex (B). (A). One representative mouse sampled from the group cohort of 8 was exposed to one of the 5 conditions listed on the left. Sagittal sections from the HC were photomicrographed at 10x. Detected molecules were informed using primary antibodies vs. pAktS473, pGSK-3βS9, β-amyloid (MoAb-2) and pTauT231 and secondary nuclear stain with DAPI are shown along the top 5 columns. To the far right is a serial section stained for brightfield with H and E. In the pTau images of column four, plaque areas are outlined in blue within which some cellular pTau signal profiles are indicated by a yellow arrowhead. WT mice had no

Aβ or pTau signals. WT,CD (wild type litter mates, control (standard) diet); TG,CD/veh (transgenic, control diet, with added vehicle); TG, CD/PS48 (transgenic, fed PS48; TG, HFD (high fat diet). (**B**). As per A, a single representative section is stained for each of the molecules above (columns 1-4), for nucleus (column 5) and H and E (column 6). Each row is from a single animal representing the group as indicated left. The apparent hypocellularity in the TG/HFD group on H and E may be a perfusion artifact related to a HFD change in vascular compliance.

Supplement Fig. 3

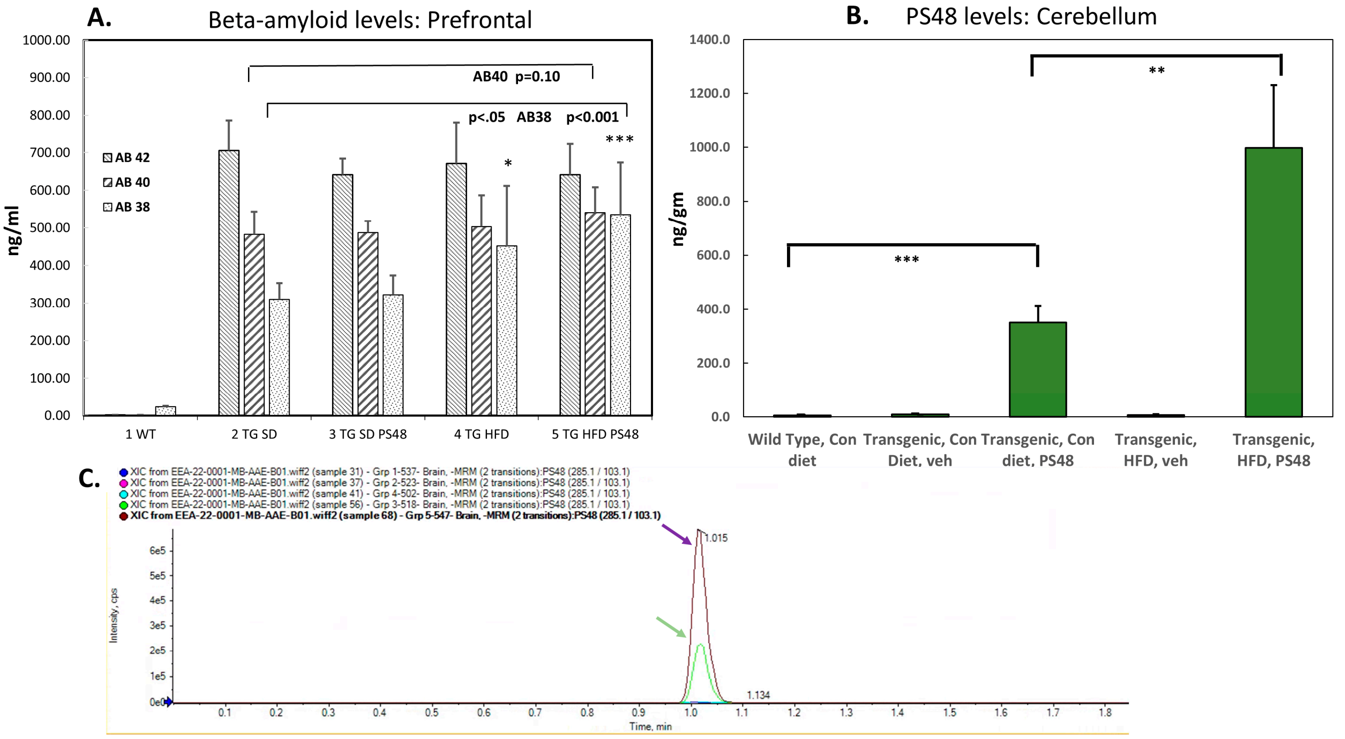

Supplement Fig. 3D

ß-amyloid plaque quantification : Moab2 anti-pan Aß

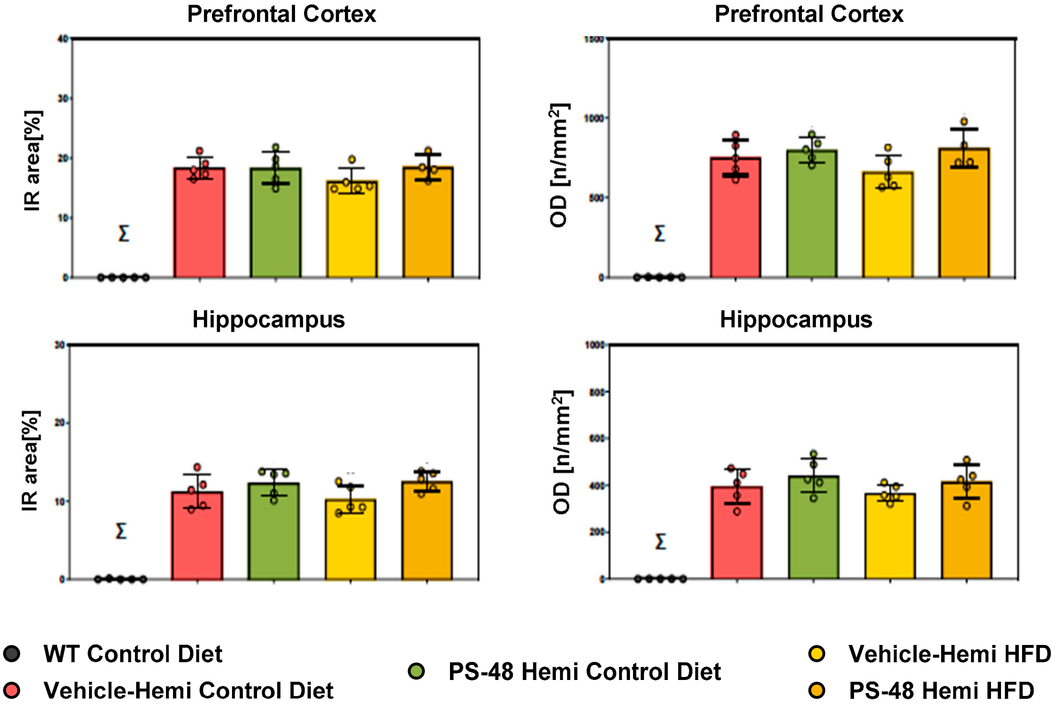

Figure S3. β-amyloid and PS48 quantification. (A). Aβ peptide concentrations in PFC homogenates were determined by ELISA and reported as ng/ml for Aβ42, 40 and 38 species. WT mice had undetectable 42 and 40 levels. TG across all groups had uniformly increased levels, ordered as Aβ42 >40 >38, without significant differences between them for Aβ42 and 40 species. Aβ38 levels were elevated under HFD conditions relative to SD ($p<0.05$, $p<0.001$). PS48 did not affect Aβ levels (n=7 per group). Results shown are combined from 2 separate experiments, each conducted in duplicate. (**B).** PS48 levels in brain sample homogenates from Cerebellum. Detectible PS48 was only found in animals fed drug (bar 3 and 5). Brain levels were higher in mice on HFD than on SD. (**$p<0.005$, *** $p<0.0001$, $\pm$ 1SD). (**C).** Mass spectrometry. Representative spectrograph shown for animal tag no. 547 (group 5, HFD/PS48, purple) and group 3 (SD/PS48, green). (**D).** β-amyloid plaque quantification. MoAb2 antibody is directed against pan Aβ species. IR and OD variables are shown. All TG groups had similar Aβ levels by this measure, consistent with (A) above.
